# Dietary Inflammatory Index and Risk of Asthenozoospermia: A Hospital-Based Case-Controlled Study in China

**DOI:** 10.3389/fnut.2021.706869

**Published:** 2021-07-29

**Authors:** Fang-Hua Liu, Xiao-Bin Wang, Zhao-Yan Wen, Han-Yuan Wang, Meng Zhang, Shuang Zhang, Yu-Ting Jiang, Jia-Yu Zhang, Hui Sun, Bo-Chen Pan, Qi-Jun Wu

**Affiliations:** ^1^Clinical Research Center, Shengjing Hospital of China Medical University, Shenyang, China; ^2^Department of Clinical Epidemiology, Shengjing Hospital of China Medical University, Shenyang, China; ^3^Center of Reproductive Medicine, Shengjing Hospital of China Medical University, Shenyang, China

**Keywords:** asthenozoospermia, case-controlled study, dietary inflammatory index, nutrients, China

## Abstract

**Background:** Evidence of associations between a pro-inflammatory diet and asthenozoospermia risk is limited. We therefore performed a case-controlled study to investigate associations between pro-inflammatory diet using dietary inflammatory index (DII) scores and asthenozoospermia risk in China.

**Methods:** Our hospital-based case-controlled study comprised 549 incident asthenozoospermia men and 581 healthy controls. All were interviewed at the infertility clinic in Shengjing Hospital of China Medical University from June 2020 to December 2020. DII scores were calculated based on dietary intake which were assessed using a validated food frequency questionnaire. Semen parameters were analyzed according to World Health Organization guidelines. An unconditional logistic regression model was used to estimate odds ratios (ORs) and corresponding 95% confidence intervals (CIs) for asthenozoospermia risk. The lowest tertile served as the reference category for regression analyses.

**Results:** After adjustment for age in the primary multivariable model, we failed to determine a significant negative association between DII and asthenozoospermia risk (for the highest tertile of DII scores compared to the lowest tertile) (OR = 0.77, 95% CI: 0.57–1.03). Similarly, non-significant associations were also identified in the multivariable model after adjusting for more potential confounders (OR = 0.86; 95% CI: 0.58–1.27). Additionally, in subgroup analyses stratified by age, body mass index, and smoking status, non-significant results were consistent with the main findings.

**Conclusions:** To our knowledge, this is the first study exploring this particular topic. Our research does not support an association between DII scores and asthenozoospermia risk. Further prospective studies with more DII relevant foods and nutrients are warranted to confirm our findings.

## Introduction

Globally, infertility is a serious public health issue affecting ~15% of couples of reproductive age (1, 2). Notably, male factors account for more than half of all infertility cases (1, 2). As a major pathological indicator of male infertility, asthenozoospermia has received considerable attention in recent years. A retrospective study showed that 19% of infertile males exhibited isolated asthenozoospermia and 63% of infertile males displayed asthenozoospermia associated with oligo- and/or teratozoo-spermia (3). According to a recent Chinese study, the proportion of patients with asthenozoospermia showed an upward trend, accounting for 50.4% of 38,905 male infertility cases between 2008 and 2016 (4). Equally, emerging evidence has also suggested that genetics (*DNAH17* and *ARMC2* genes) (5, 6), varicocele (7), endocrine-disrupting chemicals (8), stress (9), lifestyle factors such as smoking (10, 11), and environmental exposure (12) may also contribute to this condition.

Diet is a major regulator of inflammation and plays important roles in asthenozoospermia occurrence and development (13). Epidemiological studies have shown that adherence to a Western diet pattern is potentially an unfavorable indicator for asthenozoospermia risk (13), whereas Mediterranean dietary patterns are beneficial for sperm motility (14). Western dietary patterns, characterized by a high intake of refined grains, high-fat dairy products, and red meat are associated with a pro-inflammatory state (15, 16). In contrast, Mediterranean dietary patterns, characterized by a high intake of green vegetables, fish, whole grains, and fruits, and a low intake of red meat and butter, moderate olive oil consumption, and red wine are associated with an anti-inflammatory state (17, 18). Inflammation may be the major cause of the asthenozoospermia (19). Shukla et al. conducted a case-controlled study investigated the association between Tumor Necrosis Factor-α (TNF-α) (one of inflammatory cytokines) and male infertility in India, and showed that TNF-α were significantly higher in infertile men with asthenozoospermia compared to fertile men (20). To evaluate the potential inflammation impact from various diets, the dietary inflammatory index (DII) was initiated in 2009 and refined in 2014 (21, 22). Based on the literature, the DII accounts for six inflammatory markers; TNF-α, C-reactive protein, interleukin (IL)-4, IL-6, IL-10, and IL-1β, which are related to dietary factors, such as flavonoids, vitamins, macronutrients, minerals, and specific food items (21–23). Currently, several epidemiological studies have explored relationships between DII and chronic disease, including metabolic syndrome (24), cardiovascular disease (25, 26), and cancer (27, 28). These findings have supported the hypothesis that high DII levels are detrimental to health. Surprisingly, only one cross-sectional study involving 209 healthy male college students has investigated the relationship between DII and male infertility risk; with results indicating a positive relationship was identified between DII and total sperm motility and progressive sperm motility in this cohort (29). However, the small sample size and a narrow age group (18–23 years old) may limit the study interpretation.

To provide more evidence on the relationship between DII and asthenozoospermia risk, we conducted a hospital-based case-controlled study of 549 asthenozoospermia cases and 581 healthy controls in Shenyang, Liaoning Province, China. To the best of our knowledge, ours is the first large-scale study exploring this topic.

## Materials and Methods

### Study Design and Participants

Men admitted to the infertility clinic (Shengjing Hospital of China Medical University) in Liaoning, China were selected for this case-controlled study, conducted between June 2020 and December 2020. Cases included 597 men with asthenozoospermia. According to the fifth edition of the World Health Organization laboratory manual for the examination and processing of human semen (30), asthenozoospermia is defined as “total motility” (progressive + non-progressive) of <40%, including both rapid and slow progressive motility, slow motility, and non-progressive motility. The condition is also defined as progressive motility of <32%, including both rapid, slow progressive, and sluggish motility in the same class within 60 min of ejaculation over the previous 3 months. The total number (or concentration) of spermatozoa and the percentage of morphologically normal spermatozoa is ≥ lower reference limits.

Controls comprised of 612 normozoospermic men (≥15 × 10^6^ sperm/mL, ≥40% total motility, ≥32% progressive motility, and ≥4% normal forms) from infertile couples, admitted to the same infertility clinic with cases group. Patients with a varicocele history were excluded. All participants were interviewed face-to-face by trained professional interviewers. Finally, 549 men with asthenozoospermia (92%) and 581 controls (95%) completed a validated food frequency questionnaire (FFQ). The research protocol was approved by the ethics committee of Shengjing Hospital of China Medical University.

### Semen Collection and Analysis

Within the required 3–7 days abstinence period, semen samples were collected by masturbation into a plastic tube in a dedicated room. Condoms and lubricants were prohibited. Samples were allowed to liquefy for 45–60 min before analysis. Sperm is classified into four degrees (A, B, C, D) according to motility by World Health Organization. Ejaculate volume was measured and other parameters such as pH, sperm concentration, total account of sperm, total motility, and the percentage of each motile sperm grade were measured using a WLJY9000 instrument. Flow cytometry was used to assess sperm DNA fragmentation and sperm DNA staining. Pasteurization was used for sperm smear observations and morphology was assessed using an optical microscope. Normal sperm reference values were determined using World Health Organization criteria (30). All analyses were conducted by an experienced technician, and throughout the study, an external quality control was performed.

### Data Collection

Face-to-face interviews were conducted by trained interviewers at baseline using a validated questionnaire which collected general demographic characteristics, e.g., health care products (vitamin, zinc, calcium, et al.) and dietary history, personal lifestyle habits, sleep conditions, physical activity, mental condition, passive and indoor smoking, disease history, and family history of chronic disease. Simultaneously, physical examination data were collected, including height, weight [for body mass index (BMI) calculations], waist circumference, hip circumference, and blood pressure.

### Exposure Assessment

Dietary data derived FFQs were used to calculate DII scores for all participants. The DII literature consists of all qualifying publications between 1950 and 2010 reporting one or more associations between dietary components and the following inflammatory markers: IL-1β, IL-4, IL-6, IL-10, TNF-α, and C-reactive protein (22). In this study, we scored the inflammatory potential for each food parameter according to whether it increased inflammatory or decreased anti-inflammatory factors (+1), decreased inflammatory or increased anti-inflammatory factors (−1), or had no effect (0) on inflammatory or anti-inflammatory factors. In the present study, the available components that were used for calculating DII included the following nutrients: energy, carbohydrates, fiber, protein, fat, saturated fatty acids, polyunsaturated fatty acids, monounsaturated fatty acids, anthocyanin, isoflavone, cholesterol, vitamin B12, vitamin B6, beta-carotene, folic acid, niacin, riboflavin, thiamin, vitamin A, vitamin C, vitamin E, zinc, selenium, iron, magnesium, garlic, ginger, onion, pepper and tea. For each participant, in order to compute a z-score, we subtracted the “standard global mean” from the quantity of food items consumed by each subject and divided this by the “global standard deviation” (22). Then, we transformed this value into a centered percentile score to reduce skewness. Finally, we summed DII scores from all foods to calculate an overall DII score. Higher DII scores indicated more a pro-inflammatory diet whereas lower DII scores represented an anti-inflammatory diet.

### Statistical Analysis

Descriptive statistics were performed to profile and compare participant characteristics and food and nutrient intakes between groups. Continuous variables were reported using the mean ± standard deviation, and categorical variables were represented by counts and percentages. To compare differences between groups, independent sample Student *t*-tests were conducted for continuous variables and the Chi-square test for categorical variables.

Unconditional logistic regression analyses were used to estimate odds ratios (ORs) with 95% confidence intervals (CIs) to assess associations between DII scores and asthenozoospermia risk. All DII scores were divided into three categories (tertiles), and the lowest tertile of DII scores was considered the reference group. In the first regression model, we only adjusted for age (years). In the second regression model, we further adjusted for BMI (kg/m^2^), smoking status (yes/no), household income (RMB; thousand yuan), abstinence time (days), educational level (junior secondary or below, senior high school/technical secondary school, junior college/university or above), and physical activity (metabolic equivalent/hours/week). In addition, we calculated adjusted risk estimates for asthenozoospermia by DII score in subgroup analyses stratified by age, BMI, and smoking status. All analyses were conducted using SAS version 9.4 (SAS Institute Inc., Cary, NC, USA). All statistical tests were two-sided and statistical significance was set at *P* < 0.05.

## Results

The general distribution characteristics of asthenozoospermia between groups are shown (Table 1). Asthenozoospermia cases tended to be slightly older and experience a longer abstinence time than controls. In terms of semen parameters, asthenozoospermia cases had significantly lower sperm concentrations, total sperm counts, progress motility, total motility, and normal sperm morphology than controls.

**Table 1:** General participant characteristics.

**Characteristics**	**Asthenozoospermia**	**Normal**	***P*-value**
No. of participants	549	581	
Age (years)	33.29 ± 5.28	32.11 ± 4.50	<0.05
Body mass index (kg/m^2^)	26.41 ± 4.42	26.25 ± 4.55	0.55
Physical activity (MET/hours/week)	166.88 ± 103.42	165.89 ± 101.80	0.87
Television watching (hours/week)	6.52 ± 8.79	6.19 ± 8.01	0.52
Computer using (hours/week)	24.81 ± 15.95	24.90 ± 15.07	0.93
Abstinence time (days)	4.47 ± 1.48	4.28 ± 1.39	<0.05
**Semen parameters**			
Ejaculate volume (ml)	3.61 ± 1.47	3.45 ± 1.26	0.05
Sperm concentration (10^6^/ml)	58.74 ± 36.13	71.10 ± 39.88	<0.05
Total sperm count (10^6^/ml)	198.79 ± 126.04	232.18 ± 133.50	<0.05
Progress motility (%)	22.02 ± 8.72	44.58 ± 9.33	<0.05
Total motility (%)	27.94 ± 10.92	54.94 ± 11.35	<0.05
Normal sperm morphology (%)	5.70 ± 2.55	6.68 ± 2.72	<0.05
**Smoke status (** ***n*** **, %)**			0.11
No	285 (51.91)	274 (47.16)	
Yes	264 (48.09)	307 (52.84)	
**Educational level (** ***n*** **, %)**			0.67
Junior secondary or below	121 (22.04)	141 (24.27)	
Senior high school/technical secondary school	79 (14.39)	82 (14.11)	
Junior college/university or above	349 (63.57)	358 (61.62)	
**Annual family income (RMB thousand yuan), (** ***n*** **, %)**			0.76
<50	98 (17.85)	94 (16.18)	
50 to <100	209 (38.07)	226 (38.90)	
≥100	242 (44.08)	261 (44.92)	

*MET, metabolic equivalent*.

*All continuous variables are represented as the mean ± standard deviation. All categorical variables are represented as counts and percentages. P-values were derived from Student t-tests and Chi-square tests. All statistical tests are two sided*.

Food and nutrient intake distributions across groups are also shown (Table 2). When compared with controls, cases displayed higher carbohydrate consumption, however, meat consumption in cases was significantly (*P* < 0.05) lower than controls. Also, dietary intakes of study participants across the DII score tertiles are presented in Table 2. Significantly decreasing trends were observed for the food and nutrient intake (*P* < 0.05).

**Table 2:** Dietary intake of participants.

**Diets**	**Asthenozoospermia**	**Normal**	***P*-value**	**Dietary inflammatory index tertiles^a^**			***P*-value**
				**T1**	**T2**	**T3**	
No. of participants	549	581		397	390	343	
Energy (kcal/d)	1,822.70 ± 579.89	1,769.09 ± 562.01	0.11	2,270.50 ± 560.57	1,714.40 ± 336.46	1,336.74 ± 322.12	<0.05
**Nutrients**							
Proteins (g/d)	75.34 ± 25.93	73.32 ± 25.30	0.19	96.28 ± 24.65	70.50 ± 14.63	53.19 ± 13.97	<0.05
Fat (g/d)	51.14 ± 21.00	49.41 ± 20.25	0.16	65.57 ± 20.75	48.18 ± 14.26	34.86 ± 12.62	<0.05
Carbohydrates (g/d)	260.01 ± 82.29	248.86 ± 80.69	<0.05	315.05 ± 85.20	241.48 ± 55.54	198.50 ± 49.87	<0.05
Total dietary fiber (g/d)	17.70 ± 9.28	16.67 ± 8.90	0.05	25.76 ± 9.45	14.68 ± 3.76	10.05 ± 3.16	<0.05
Cholesterol (g/d)	402.91 ± 219.97	379.58 ± 203.83	0.06	510.79 ± 227.49	369.69 ± 171.73	276.30 ± 156.87	<0.05
SFA (g/d)	15.44 ± 6.63	15.33 ± 6.76	0.78	19.41 ± 6.96	15.02 ± 5.22	11.14 ± 4.87	<0.05
MUFA (g/d)	15.76 ± 6.77	15.56 ± 6.58	0.60	20.13 ± 6.97	15.10 ± 4.81	11.11 ± 4.48	<0.05
PUFA (g/d)	7.33 ± 3.75	6.92 ± 3.37	0.06	9.88 ± 3.92	6.66 ± 2.15	4.44 ± 1.61	<0.05
Thiamine (mg/d)	0.83 ± 0.43	0.84 ± 0.43	0.65	1.11 ± 0.44	0.80 ± 0.36	0.57 ± 0.28	<0.05
Riboflavin (mg/d)	1.11 ± 0.44	1.06 ± 0.44	0.08	1.46 ± 0.44	1.00 ± 0.24	0.74 ± 0.24	<0.05
Niacin (mg/d)	19.18 ± 5.53	19.38 ± 5.61	0.55	23.16 ± 5.48	18.75 ± 4.05	15.41 ± 4.01	<0.05
Vitamin B6 (mg/d)	0.57 ± 0.24	0.55 ± 0.23	0.22	0.77 ± 0.23	0.50 ± 0.12	0.37 ± 0.12	<0.05
β-carotene (μg/d)	1,262.38 ± 982.35	1,204.13 ± 924.92	0.30	1,958.29 ± 1,140.38	971.35 ± 571.66	689.15 ± 370.21	<0.05
Total Vitamin A (μgRE/d)	658.63 ± 599.51	616.36 ± 610.62	0.24	1,016.40 ± 811.67	513.67 ± 328.07	337.75 ± 224.04	<0.05
Total Vitamin E (mg/d)	16.40 ± 9.91	15.40 ± 9.48	0.08	23.86 ± 10.48	14.38 ± 5.58	8.36 ± 3.80	<0.05
Vitamin C (mg/d)	109.07 ± 69.05	108.40 ± 69.86	0.87	168.88 ± 72.73	93.00 ± 40.07	56.98 ± 28.23	<0.05
Vitamin B12 (μg/d)	0.20 ± 0.20	0.20 ± 0.23	0.98	0.26 ± 0.22	0.21 ± 0.23	0.12 ± 0.16	<0.05
Folic acid (μg/d)	240.14 ± 130.77	226.28 ± 129.26	0.07	357.06 ± 135.11	198.10 ± 53.00	129.13 ± 38.55	<0.05
**Food groups**							
Milk (g/d)	118.22 ± 124.14	106.55 ± 114.29	0.10	155.07 ± 144.95	100.24 ± 98.99	76.26 ± 88.85	<0.05
Meat (g/d)	99.81 ± 45.88	106.71 ± 49.02	<0.05	116.07 ± 48.72	105.98 ± 44.08	85.66 ± 44.94	<0.05
Fish (g/d)	22.94 ± 19.69	22.84 ± 20.49	0.93	30.79 ± 24.20	22.18 ± 17.41	14.55 ± 12.94	<0.05
Vegetable (g/d)	206.13 ± 141.97	193.98 ± 130.17	0.13	314.00 ± 154.22	161.86 ± 74.79	111.02 ± 50.65	<0.05
Fruit (g/d)	173.55 ± 194.54	167.24 ± 171.60	0.56	273.27 ± 253.30	139.24 ± 98.20	86.44 ± 69.95	<0.05
Legumes and nuts (mg/d)	106.47 ± 85.90	99.39 ± 84.51	0.16	159.95 ± 98.35	92.32 ± 62.08	48.66 ± 39.21	<0.05
Tea and coffee (mg/d)	180.03 ± 316.46	160.79 ± 301.52	0.30	246.47 ± 374.95	173.73 ± 302.56	77.71 ± 180.84	<0.05
Alcohol (mg/d)	139.50 ± 235.97	166.95 ± 258.18	0.06	201.42 ± 272.92	159.47 ± 251.95	91.61 ± 194.66	<0.05

*MUFA, monounsaturated fatty acids; PUFA, polyunsaturated fatty acids; SFA, saturated fatty acids*.

a *Tertile DII score range: T1 (<0.65), T2 (0.65 to <2.32), and T3 (≥2.32)*.

*All continuous variables are shown as the mean and standard deviation*.

*All statistical tests are two sided*.

Associations between DII scores and asthenozoospermia risk are shown (Table 3). We observed a significant negative association between DII score and the odds of asthenozoospermia in the model 1 (OR = 0.74, 95% CI: 0.55–0.98). However, in model 2 which adjusted for age only, the highest DII tertile was not associated with asthenozoospermia risk (OR = 0.77, 95% CI: 0.57–1.03) when compared with the lowest tertile. Similar results were also found (OR_T3vs.T1_ = 0.86, 95% CI: 0.58–1.27) when further adjusted for BMI, smoking status, household income, total energy intake, abstinence time, educational level, and physical activity. In addition, we evaluated the effect-modifying roles of age, BMI, and smoking status. Not surprisingly, the results of these subgroup analyses were agreed with the main findings (Table 4).

**Table 3:** Adjusted risk estimates for asthenozoospermia by dietary inflammatory index.

	**Dietary inflammatory index tertiles**			***P* for trend**
	**T1**	**T2**	**T3**	
DII score range	<0.65	0.65 to <2.32	≥2.32	
Cases/Controls	203/193	195/193	151/195	
Model 1^a^	1.00 (Ref)	0.96 (0.73–1.27)	0.74 (0.55–0.98)	0.06
Model 2^b^	1.00 (Ref)	0.97 (0.73–1.28)	0.77 (0.57–1.03)	0.10
Model 3^c^	1.00 (Ref)	1.05 (0.76–1.45)	0.86 (0.58–1.27)	0.53

*CI, confidence interval; OR, odds ratio; T, Tertiles; Ref, reference*.

a *Model 1: unadjusted*.

b *Model 2: adjusted for age*.

c *Model 3: adjusted for age, body mass index, smoking status, household income, total energy intake, abstinence time, educational level, and physical activity*.

**Table 4:** Adjusted risk estimates for asthenozoospermia by dietary inflammatory index stratified by age, body mass index, and smoking status.

**Stratified**	**Cases**	**Controls**	**Dietary inflammatory index tertiles**			***P* for trend**	***P* for interaction**
			**T1**	**T2**	**T3**		
Age (years)^a^							0.34
<32	221	276	1.00 (Ref)	0.88 (0.53–1.45)	0.80 (0.45–1.44)	0.46	
≥32	328	305	1.00 (Ref)	1.12 (0.73–1.73)	0.93 (0.55–1.58)	0.86	
BMI (kg/m^2^)^b^							0.18
<25	200	239	1.00 (Ref)	0.91 (0.53–1.56)	0.61 (0.32–1.16)	0.25	
≥25	349	342	1.00 (Ref)	1.08 (0.72–1.64)	1.03 (0.62–1.69)	0.86	
Smoking status^c^							0.27
Smokers	285	274	1.00 (Ref)	1.11 (0.74–1.65)	1.17 (0.75–1.84)	0.62	
Never smokers	264	307	1.00 (Ref)	1.08 (0.68–1.71)	0.86 (0.49–1.50)	0.65	

*CI, confidence interval; OR, odds ratio; T, Tertiles; Ref, reference*.

a *Stratified 1: stratified by age. Adjusted for body mass index, smoking status, household income, total energy intake, abstinence time, educational level, and physical activity. When age <32, the tertile DII score range is T1 (<0.87), T2 (0.87 to <2.34), and T3 (≥2.34). When age ≥32, the tertile DII score range is T1 (<0.53), T2 (0.53 to <2.15), and T3 (≥2.15)*.

b *Stratified 2: stratified by body mass index. Adjusted for age, smoking status, household income, total energy intake, abstinence time, educational level, and physical activity. When body mass index <25, the tertile DII score range is T1 (<0.65), T2 (0.65 to <2.36), and T3 (≥2.36). When body mass index ≥ 25, tertile DII score range is T1 (<0.64), T2 (0.64 to <2.20), and T3 (≥2.20)*.

c *Stratified 3: stratified by smoking status. Adjusted for age, body mass index, household income, total energy intake, abstinence time, educational level, and physical activity. For smokers, the tertile DII score range is T1 (<0.65), T2 (0.65 to <2.37), and T3 (≥2.37). For non-smokers, the tertile DII score range is T1 (<0.56), T2 (0.56 to <2.21), and T3 (≥2.21)*.

## Discussion

To the best of our knowledge, ours is the first study to investigate the relationship between a pro-inflammatory diet, measured the DII scores, and asthenozoospermia risk. In this hospital-based case-controlled study, although the point estimate of multivariable regression analyses indicated a negative association between DII scores and asthenozoospermia risk, we observed no statistically significant findings. It might be lack of common components that used to calculate DII (such as n-3 fatty acids, n-6 fatty acids and vitamin D). However, we failed to get the information from the FFQ used in current investigation. Moreover, in a hospital-based case-controlled study, recall capabilities and selection bias may influence the accuracy of exposure.

DII studies in asthenozoospermia are scarce. A cross-sectional Spanish study explored the relationship between DII and male reproductive parameters (29). This study in 209 healthy male university students reported that a high DII was positively associated with total sperm motility and progressive sperm motility (29). These Spanish data were inconsistent with our findings, which may be attributed to different participants, habits, outcomes and sample size. Participants included in our study aged 18–79 years, however, the participants of Spanish research are college students and age of population is narrow. In addition, Spain belongs to the Mediterranean region, and the dietary habits may be close to the mediterranean diet which is an anti-inflammatory diet (17). Our study carried out in Liaoning, China and the dietary habits may be similar to the western dietary patterns of pro-inflammatory diets (15). In addition, two previous studies investigated the relationship between dietary patterns and asthenozoospermia. Eslamian et al. performed two case-controlled studies (107 asthenozoospermic men and 235 age-matched controls) to explore the association between dietary patterns and nutrient patterns and the odds of asthenozoospermia, respectively (13, 31). And their findings suggested that western pattern of pro-inflammatory state is positively correlated with asthenozoospermia and pattern comprising mainly of antioxidant nutrients of anti-inflammatory may be inversely associated with asthenozoospermia. The reason for the different results from ours may be due to different food items, such as grains, condiments, sauces and calcium included in previous two studies, but not included in our study.

A previous study showed that inflammation may affect prostate function, which in turn affects sperm quality (32). Researchers also reported increased leukocytosis, IL-1 levels, and reactive oxygen species in patients with chronic prostatitis, along with decreased sperm quality (33, 34). Although reproductive tract infections and elevated inflammatory factor levels are associated with negative consequences for male reproduction, spermatogenesis, and semen quality (35), no research has yet uncovered the precise biological mechanisms underpinning DII and asthenozoospermia, therefore future studies in this area are warranted.

This study had several strengths. First, ours was the first report to focus on associations between DII and asthenozoospermia risk. Second, our large sample size and high participation rates were advantageous in reducing random errors often associated with studies with low participant numbers. Furthermore, a validated FFQ was used as a comprehensive method to collect detailed data about habitual food intake associated with inflammatory characteristics. In addition, DII was used as a research tool to focus on the effects of the overall diet on asthenozoospermia risk, instead of individual foods or nutrients. Of note, we adjusted for several confounding factors which ensured the authenticity and reliability of our research results.

Conversely, our study also had several limitations meaning our results must be interpreted with caution. First, as in any hospital-based case-controlled study, recall capabilities and selection bias are problematic. There may have been bias in the accuracy and completeness of case recall of exposure history in the year prior to their diagnosis. Similarly, the control group from the hospital may have made different dietary choices when compared with the general population (36). To improve subject comparability, we used a highly reproducible validated FFQ and selected highly trained and skilled researchers to collect dietary information (37, 38). Second, as observed in previous studies (39), we lacked data on some dietary DII components, including flavonoids, turmeric, rosemary, saffron, and thyme, which were not calculated for DII scores; however, as they are uncommon ingredients in Chinese diets, they may have had no significant effects on DII scores. Third, although the FFQ is commonly used to assess food intake in epidemiological studies, measurement bias remains a possibility. Based on standard food portions to calculate food intake, we were unable to determine the precise food intake of participants. Thus, associations between DII and asthenozoospermia risk may be underestimated. To reduce bias effects, energy adjustments were applied to our analyses. Fourthly, although a diverse array of potential confounders was adjusted for in this research, residual confounders cannot be completely excluded, including endocrine-disrupting chemicals (8) and ambient air pollution (40). Future studies should aim to eliminate these issues and clarify the relationship between exposure and outcomes.

In conclusion, our data suggested DII was not associated with asthenozoospermia risk. Future studies must include more food parameters to comprehensively calculate DII scores. Equally, large prospective studies should be designed and implemented to validate our results or identify precise associations between the diet and asthenozoospermia.

## Data Availability Statement

The original contributions presented in the study are included in the article/supplementary material, further inquiries can be directed to the corresponding author/s.

## Ethics Statement

The studies involving human participants were reviewed and approved by the ethics committee of Shengjing Hospital of China Medical University. The patients/participants provided their written informed consent to participate in this study.

## Author Contributions

Q-JW and B-CP conceived the study. F-HL, X-BW, and Z-YW contributed to the design. F-HL, Z-YW, H-YW, and MZ collected the data. SZ and J-YZ cleaned the data and checked the discrepancy. F-HL and SZ analyzed the data. Y-TJ, J-YZ, and HS interpreted the data. All authors interpreted the data, read the manuscript, and approved the final vision.

## Conflict of Interest

The authors declare that the research was conducted in the absence of any commercial or financial relationships that could be construed as a potential conflict of interest.

## Publisher's Note

All claims expressed in this article are solely those of the authors and do not necessarily represent those of their affiliated organizations, or those of the publisher, the editors and the reviewers. Any product that may be evaluated in this article, or claim that may be made by its manufacturer, is not guaranteed or endorsed by the publisher.
